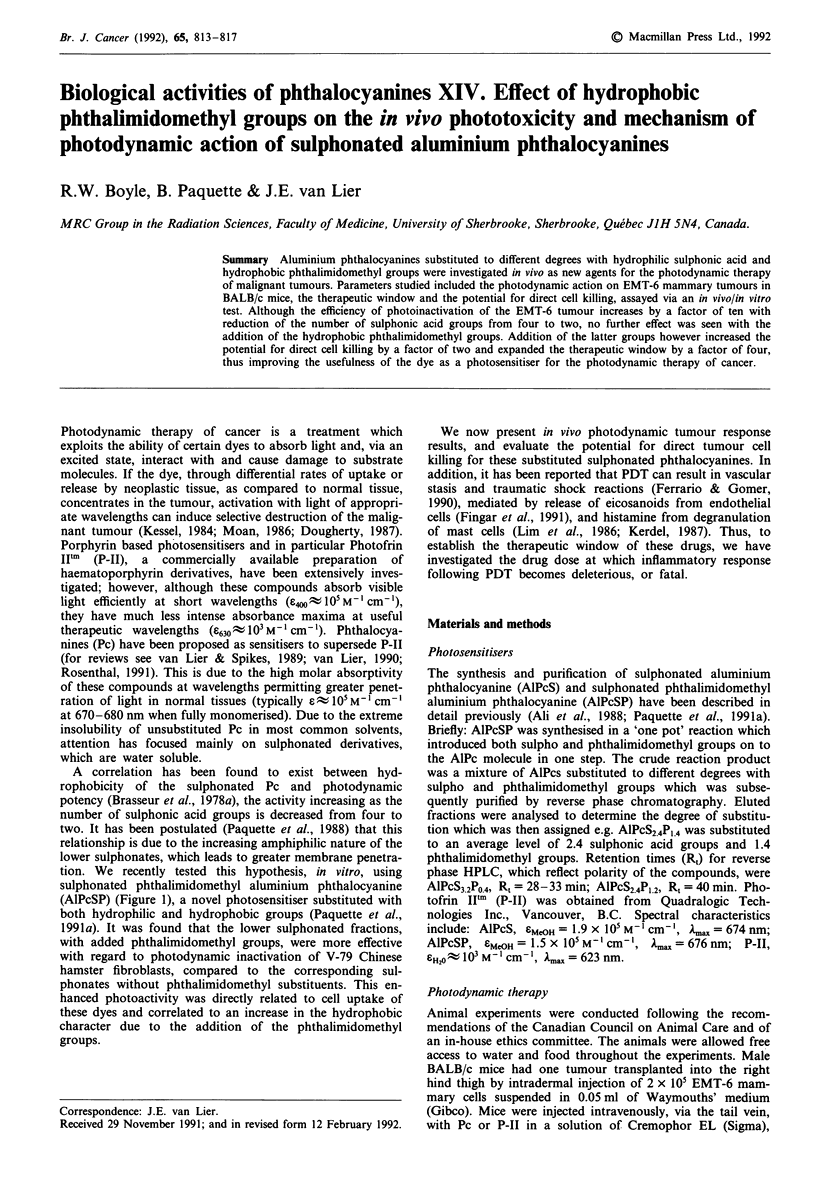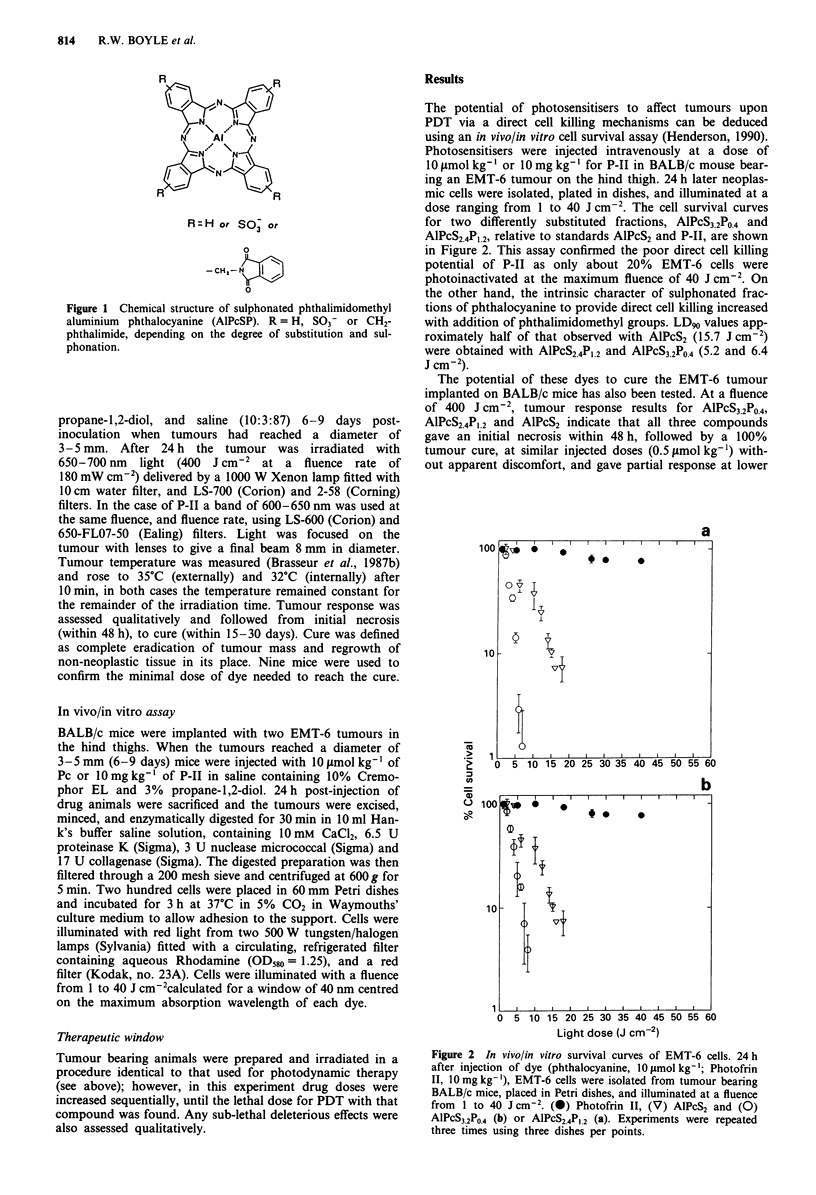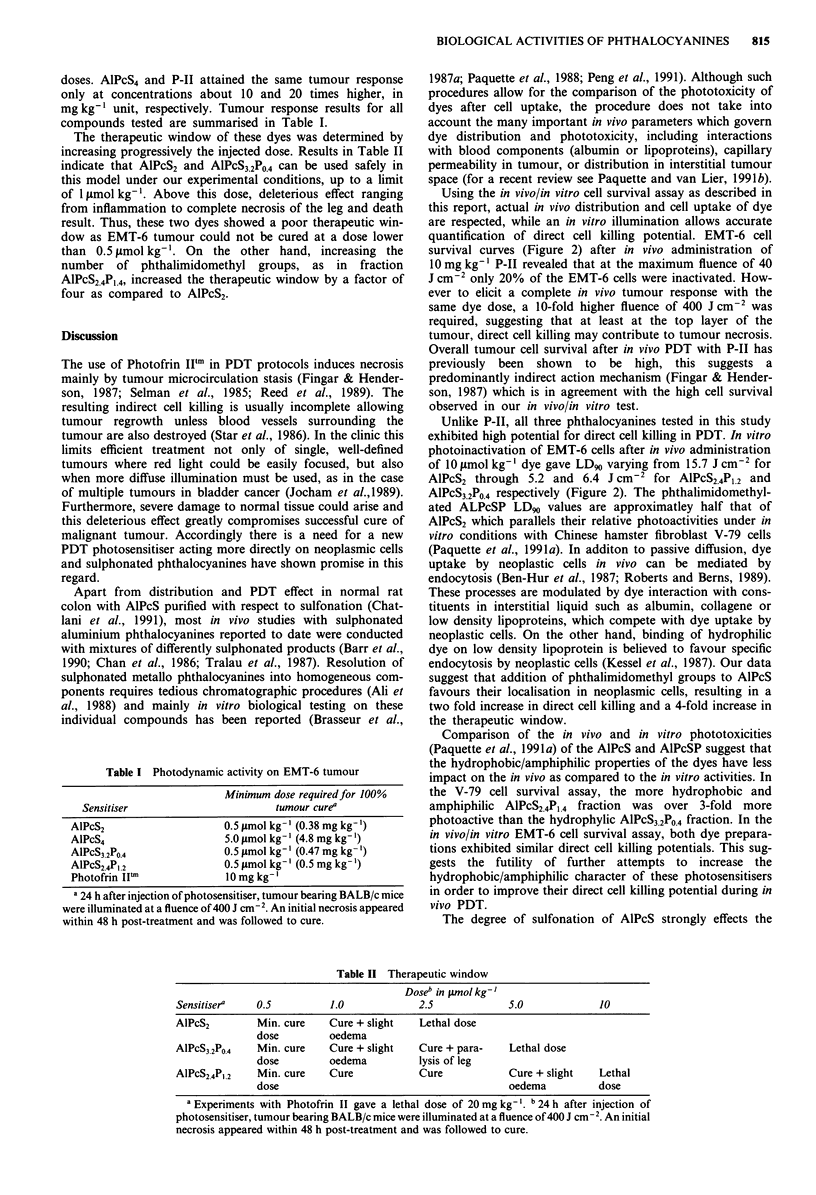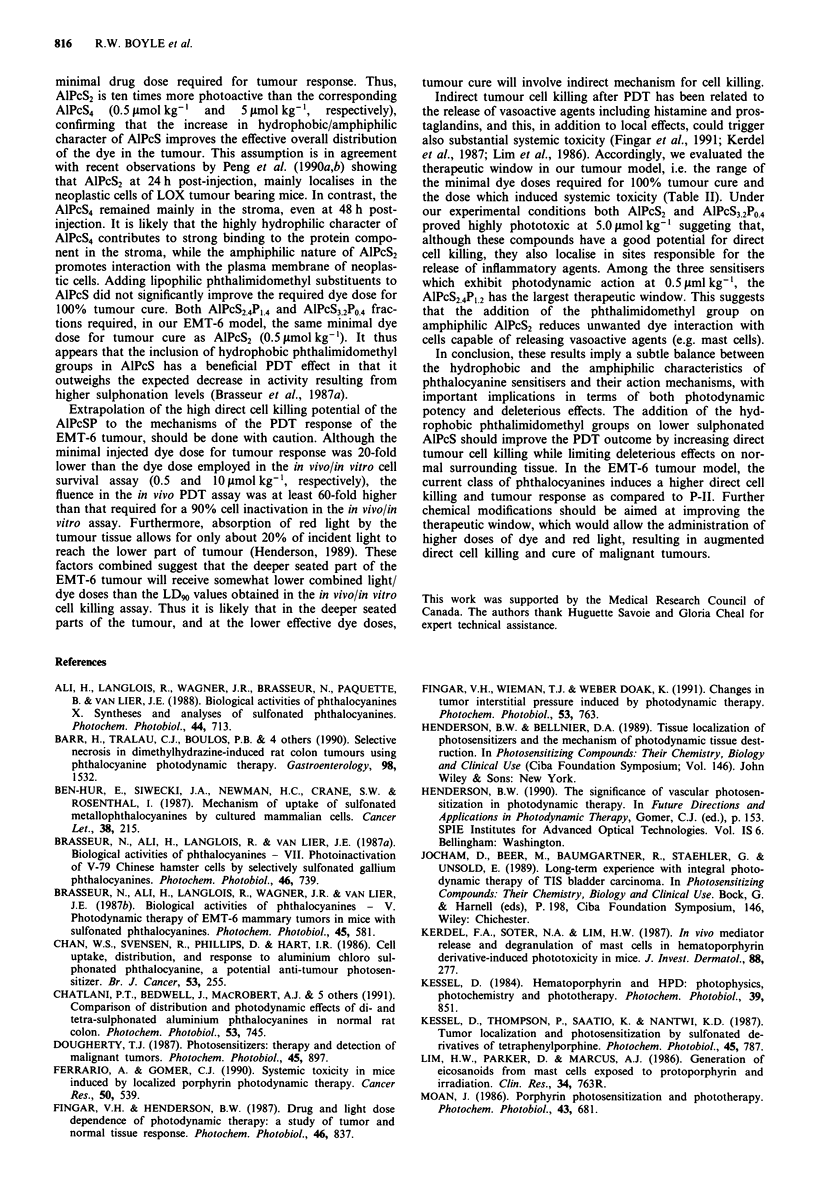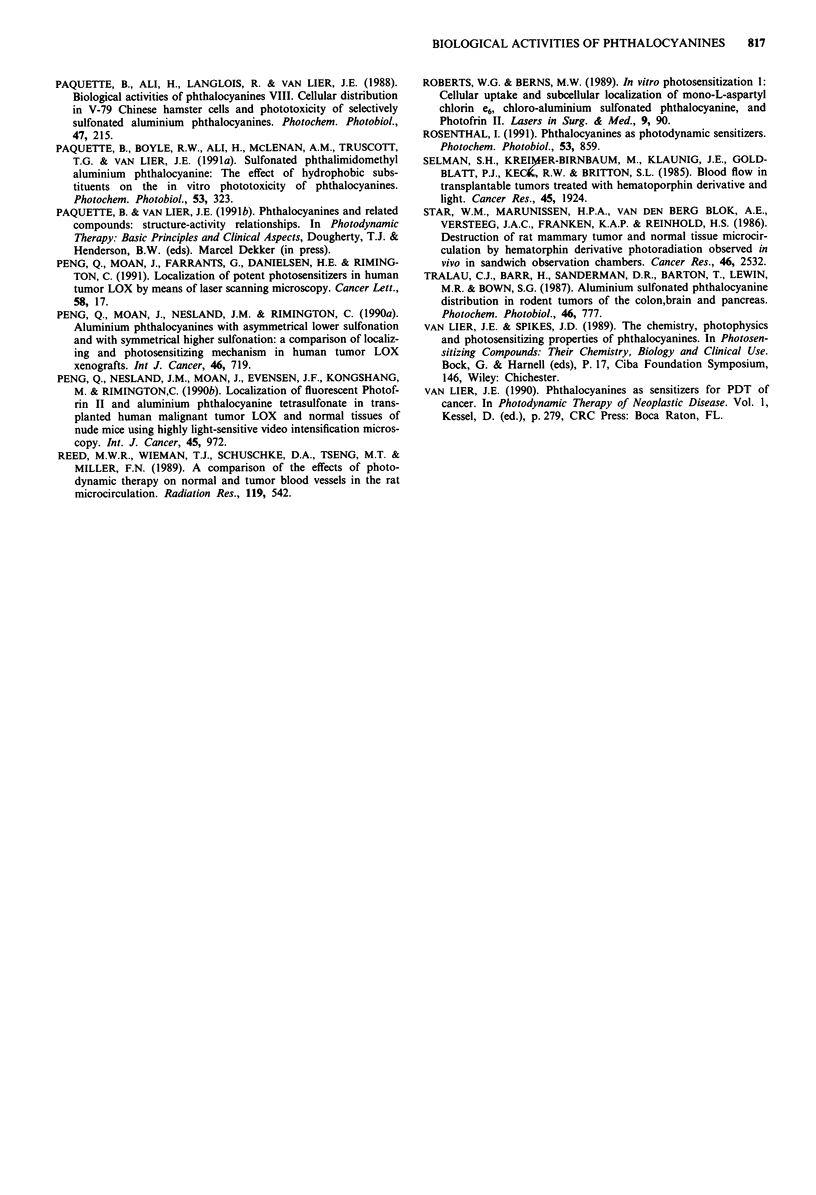# Biological activities of phthalocyanines. XIV. Effect of hydrophobic phthalimidomethyl groups on the in vivo phototoxicity and mechanism of photodynamic action of sulphonated aluminium phthalocyanines.

**DOI:** 10.1038/bjc.1992.174

**Published:** 1992-06

**Authors:** R. W. Boyle, B. Paquette, J. E. van Lier

**Affiliations:** MRC Group in the Radiation Sciences, Faculty of Medicine, University of Sherbrooke, Québec, Canada.

## Abstract

Aluminium phthalocyanines substituted to different degrees with hydrophilic sulphonic acid and hydrophobic phthalimidomethyl groups were investigated in vivo as new agents for the photodynamic therapy of malignant tumours. Parameters studied included the photodynamic action on EMT-6 mammary tumours in BALB/c mice, the therapeutic window and the potential for direct cell killing, assayed via an in vivo/in vitro test. Although the efficiency of photoinactivation of the EMT-6 tumour increases by a factor of ten with reduction of the number of sulphonic acid groups from four to two, no further effect was seen with the addition of the hydrophobic phthalimidomethyl groups. Addition of the latter groups however increased the potential for direct cell killing by a factor of two and expanded the therapeutic window by a factor of four, thus improving the usefulness of the dye as a photosensitiser for the photodynamic therapy of cancer.


Br. J. Cancer (1992), 65, 813-817 © Macmillan Press Ltd., 1992

### Biological activities of phthalocyanines XIV. Effect of hydrophobic phthalimidomethyl groups on the *in vivo* phototoxicity and mechanism of photodynamic action of sulphonated aluminium phthalocyanines

R.W. Boyle, B. Paquette & J.E. van Lier

*MRC Group in the Radiation Sciences, Faculty of Medicine, University of Sherbrooke, Sherbrooke, Québec J1H 5N4, Canada.*

**Summary** Aluminium phthalocyanines substituted to different degrees with hydrophilic sulphonic acid and hydrophobic phthalimidomethyl groups were investigated *in vivo* as new agents for the photodynamic therapy of malignant tumours. Parameters studied included the photodynamic action on EMT-6 mammary tumours in BALB/c mice, the therapeutic window and the potential for direct cell killing, assayed via an *in vivo/in vitro* test. Although the efficiency of photoinactivation of the EMT-6 tumour increases by a factor of ten with reduction of the number of sulphonic acid groups from four to two, no further effect was seen with the addition of the hydrophobic phthalimidomethyl groups. Addition of the latter groups however increased the potential for direct cell killing by a factor of two and expanded the therapeutic window by a factor of four, thus improving the usefulness of the dye as a photosensitiser for the photodynamic therapy of cancer.

Photodynamic therapy of cancer is a treatment which exploits the ability of certain dyes to absorb light and, via an excited state, interact with and cause damage to substrate molecules. If the dye, through differential rates of uptake or release by neoplastic tissue, as compared to normal tissue, concentrates in the tumour, activation with light of appropriate wavelengths can induce selective destruction of the malignant tumour (Kessel, 1984; Moan, 1986; Dougherty, 1987). Porphyrin based photosensitisers and in particular Photofrin II[tm] (P-II), a commercially available preparation of haematoporphyrin derivatives, have been extensively investigated; however, although these compounds absorb visible light efficiently at short wavelengths ($\varepsilon_{400} \approx 10^5\,M^{-1}\,cm^{-1}$), they have much less intense absorbance maxima at useful therapeutic wavelengths ($\varepsilon_{630} \approx 10^3\,M^{-1}\,cm^{-1}$). Phthalocyanines (Pc) have been proposed as sensitisers to supersede P-II (for reviews see van Lier & Spikes, 1989; van Lier, 1990; Rosenthal, 1991). This is due to the high molar absorptivity of these compounds at wavelengths permitting greater penetration of light in normal tissues (typically $\varepsilon \approx 10^5\,M^{-1}\,cm^{-1}$ at 670-680 nm when fully monomerised). Due to the extreme insolubility of unsubstituted Pc in most common solvents, attention has focused mainly on sulphonated derivatives, which are water soluble.

A correlation has been found to exist between hydrophobicity of the sulphonated Pc and photodynamic potency (Brasseur *et al.*, 1978*a*), the activity increasing as the number of sulphonic acid groups is decreased from four to two. It has been postulated (Paquette *et al.*, 1988) that this relationship is due to the increasing amphiphilic nature of the lower sulphonates, which leads to greater membrane penetration. We recently tested this hypothesis, *in vitro*, using sulphonated phthalimidomethyl aluminium phthalocyanine (AlPcSP) (Figure 1), a novel photosensitiser substituted with both hydrophilic and hydrophobic groups (Paquette *et al.*, 1991*a*). It was found that the lower sulphonated fractions, with added phthalimidomethyl groups, were more effective with regard to photodynamic inactivation of V-79 Chinese hamster fibroblasts, compared to the corresponding sulphonates without phthalimidomethyl substituents. This enhanced photoactivity was directly related to cell uptake of these dyes and correlated to an increase in the hydrophobic character due to the addition of the phthalimidomethyl groups.

We now present *in vivo* photodynamic tumour response results, and evaluate the potential for direct tumour cell killing for these substituted sulphonated phthalocyanines. In addition, it has been reported that PDT can result in vascular stasis and traumatic shock reactions (Ferrario & Gomer, 1990), mediated by release of eicosanoids from endothelial cells (Fingar *et al.*, 1991), and histamine from degranulation of mast cells (Lim *et al.*, 1986; Kerdel, 1987). Thus, to establish the therapeutic window of these drugs, we have investigated the drug dose at which inflammatory response following PDT becomes deleterious, or fatal.

### Materials and methods

### *Photosensitisers*

The synthesis and purification of sulphonated aluminium phthalocyanine (AlPcS) and sulphonated phthalimidomethyl aluminium phthalocyanine (AlPcSP) have been described in detail previously (Ali *et al.*, 1988; Paquette *et al.*, 1991a). Briefly: AlPcSP was synthesised in a 'one pot' reaction which introduced both sulpho and phthalimidomethyl groups on to the AlPc molecule in one step. The crude reaction product was a mixture of AlPcs substituted to different degrees with sulpho and phthalimidomethyl groups which was subsequently purified by reverse phase chromatography. Eluted fractions were analysed to determine the degree of substitution which was then assigned e.g. $AlPcS_{2.4}P_{1.4}$ was substituted to an average level of 2.4 sulphonic acid groups and 1.4 phthalimidomethyl groups. Retention times ($R_t$) for reverse phase HPLC, which reflect polarity of the compounds, were $AlPcS_{3.2}P_{0.4}$, $R_t$ = 28-33 min; $AlPcS_{2.4}P_{1.2}$, $R_t$ = 40 min. Photofrin II[tm] (P-II) was obtained from Quadralogic Technologies Inc., Vancouver, B.C. Spectral characteristics include: AlPcS, $\varepsilon_{MeOH} = 1.9 \times 10^5\,M^{-1}\,cm^{-1}$, $\lambda_{max}$ = 674 nm; AlPcSP, $\varepsilon_{MeOH} = 1.5 \times 10^5\,M^{-1}\,cm^{-1}$, $\lambda_{max}$ = 676 nm; P-II, $\varepsilon_{H_2O} \approx 10^3\,M^{-1}\,cm^{-1}$, $\lambda_{max}$ = 623 nm.

### *Photodynamic therapy*

Animal experiments were conducted following the recommendations of the Canadian Council on Animal Care and of an in-house ethics committee. The animals were allowed free access to water and food throughout the experiments. Male BALB/c mice had one tumour transplanted into the right hind thigh by intradermal injection of $2 \times 10^5$ EMT-6 mammary cells suspended in 0.05 ml of Waymouths' medium (Gibco). Mice were injected intravenously, via the tail vein, with Pc or P-II in a solution of Cremophor EL (Sigma),

Correspondence: J.E. van Lier.
Received 29 November 1991; and in revised form 12 February 1992.

**Figure 1** Chemical structure of sulphonated phthalimidomethyl aluminium phthalocyanine (AlPcSP). R = H, $SO_3^-$ or $CH_2$-phthalimide, depending on the degree of substitution and sulphonation.

propane-1,2-diol, and saline (10:3:87) 6–9 days post-inoculation when tumours had reached a diameter of 3–5 mm. After 24 h the tumour was irradiated with 650–700 nm light (400 J $cm^{-2}$ at a fluence rate of 180 mW $cm^{-2}$) delivered by a 1000 W Xenon lamp fitted with 10 cm water filter, and LS-700 (Corion) and 2-58 (Corning) filters. In the case of P-II a band of 600–650 nm was used at the same fluence, and fluence rate, using LS-600 (Corion) and 650-FL07-50 (Ealing) filters. Light was focused on the tumour with lenses to give a final beam 8 mm in diameter. Tumour temperature was measured (Brasseur *et al.*, 1987b) and rose to 35°C (externally) and 32°C (internally) after 10 min, in both cases the temperature remained constant for the remainder of the irradiation time. Tumour response was assessed qualitatively and followed from initial necrosis (within 48 h), to cure (within 15–30 days). Cure was defined as complete eradication of tumour mass and regrowth of non-neoplastic tissue in its place. Nine mice were used to confirm the minimal dose of dye needed to reach the cure.

### In vivo/in vitro *assay*

BALB/c mice were implanted with two EMT-6 tumours in the hind thighs. When the tumours reached a diameter of 3–5 mm (6–9 days) mice were injected with 10 μmol $kg^{-1}$ of Pc or 10 mg $kg^{-1}$ of P-II in saline containing 10% Cremophor EL and 3% propane-1,2-diol. 24 h post-injection of drug animals were sacrificed and the tumours were excised, minced, and enzymatically digested for 30 min in 10 ml Hank's buffer saline solution, containing 10 mM $CaCl_2$, 6.5 U proteinase K (Sigma), 3 U nuclease micrococcal (Sigma) and 17 U collagenase (Sigma). The digested preparation was then filtered through a 200 mesh sieve and centrifuged at 600 *g* for 5 min. Two hundred cells were placed in 60 mm Petri dishes and incubated for 3 h at 37°C in 5% $CO_2$ in Waymouths' culture medium to allow adhesion to the support. Cells were illuminated with red light from two 500 W tungsten/halogen lamps (Sylvania) fitted with a circulating, refrigerated filter containing aqueous Rhodamine ($OD_{580}$ = 1.25), and a red filter (Kodak, no. 23A). Cells were illuminated with a fluence from 1 to 40 J $cm^{-2}$calculated for a window of 40 nm centred on the maximum absorption wavelength of each dye.

### *Therapeutic window*

Tumour bearing animals were prepared and irradiated in a procedure identical to that used for photodynamic therapy (see above); however, in this experiment drug doses were increased sequentially, until the lethal dose for PDT with that compound was found. Any sub-lethal deleterious effects were also assessed qualitatively.

### Results

The potential of photosensitisers to affect tumours upon PDT via a direct cell killing mechanisms can be deduced using an *in vivo/in vitro* cell survival assay (Henderson, 1990). Photosensitisers were injected intravenously at a dose of 10 μmol $kg^{-1}$ or 10 mg $kg^{-1}$ for P-II in BALB/c mouse bearing an EMT-6 tumour on the hind thigh. 24 h later neoplasmic cells were isolated, plated in dishes, and illuminated at a dose ranging from 1 to 40 J $cm^{-2}$. The cell survival curves for two differently substituted fractions, $AlPcS_{3.2}P_{0.4}$ and $AlPcS_{2.4}P_{1.2}$, relative to standards $AlPcS_2$ and P-II, are shown in Figure 2. This assay confirmed the poor direct cell killing potential of P-II as only about 20% EMT-6 cells were photoinactivated at the maximum fluence of 40 J $cm^{-2}$. On the other hand, the intrinsic character of sulphonated fractions of phthalocyanine to provide direct cell killing increased with addition of phthalimidomethyl groups. $LD_{90}$ values approximately half of that observed with $AlPcS_2$ (15.7 J $cm^{-2}$) were obtained with $AlPcS_{2.4}P_{1.2}$ and $AlPcS_{3.2}P_{0.4}$ (5.2 and 6.4 J $cm^{-2}$).

The potential of these dyes to cure the EMT-6 tumour implanted on BALB/c mice has also been tested. At a fluence of 400 J $cm^{-2}$, tumour response results for $AlPcS_{3.2}P_{0.4}$, $AlPcS_{2.4}P_{1.2}$ and $AlPcS_2$ indicate that all three compounds gave an initial necrosis within 48 h, followed by a 100% tumour cure, at similar injected doses (0.5 μmol $kg^{-1}$) without apparent discomfort, and gave partial response at lower

**Figure 2** *In vivo/in vitro* survival curves of EMT-6 cells. 24 h after injection of dye (phthalocyanine, 10 μmol $kg^{-1}$; Photofrin II, 10 mg $kg^{-1}$), EMT-6 cells were isolated from tumour bearing BALB/c mice, placed in Petri dishes, and illuminated at a fluence from 1 to 40 J $cm^{-2}$. (●) Photofrin II, (∇) $AlPcS_2$ and (O) $AlPcS_{3.2}P_{0.4}$ (**b**) or $AlPcS_{2.4}P_{1.2}$ (**a**). Experiments were repeated three times using three dishes per points.

doses. $AlPcS_4$ and P-II attained the same tumour response only at concentrations about 10 and 20 times higher, in $mg\,kg^{-1}$ unit, respectively. Tumour response results for all compounds tested are summarised in Table I.

The therapeutic window of these dyes was determined by increasing progressively the injected dose. Results in Table II indicate that $AlPcS_2$ and $AlPcS_{3.2}P_{0.4}$ can be used safely in this model under our experimental conditions, up to a limit of $1\,\mu mol\,kg^{-1}$. Above this dose, deleterious effect ranging from inflammation to complete necrosis of the leg and death result. Thus, these two dyes showed a poor therapeutic window as EMT-6 tumour could not be cured at a dose lower than $0.5\,\mu mol\,kg^{-1}$. On the other hand, increasing the number of phthalimidomethyl groups, as in fraction $AlPcS_{2.4}P_{1.4}$, increased the therapeutic window by a factor of four as compared to $AlPcS_2$.

### Discussion

The use of Photofrin II[tm] in PDT protocols induces necrosis mainly by tumour microcirculation stasis (Fingar & Henderson, 1987; Selman *et al.*, 1985; Reed *et al.*, 1989). The resulting indirect cell killing is usually incomplete allowing tumour regrowth unless blood vessels surrounding the tumour are also destroyed (Star *et al.*, 1986). In the clinic this limits efficient treatment not only of single, well-defined tumours where red light could be easily focused, but also when more diffuse illumination must be used, as in the case of multiple tumours in bladder cancer (Jocham *et al.*,1989). Furthermore, severe damage to normal tissue could arise and this deleterious effect greatly compromises successful cure of malignant tumour. Accordingly there is a need for a new PDT photosensitiser acting more directly on neoplasmic cells and sulphonated phthalocyanines have shown promise in this regard.

Apart from distribution and PDT effect in normal rat colon with AlPcS purified with respect to sulfonation (Chatlani *et al.*, 1991), most *in vivo* studies with sulphonated aluminium phthalocyanines reported to date were conducted with mixtures of differently sulphonated products (Barr *et al.*, 1990; Chan *et al.*, 1986; Tralau *et al.*, 1987). Resolution of sulphonated metallo phthalocyanines into homogeneous components requires tedious chromatographic procedures (Ali *et al.*, 1988) and mainly *in vitro* biological testing on these individual compounds has been reported (Brasseur *et al.*, 1987*a*; Paquette *et al.*, 1988; Peng *et al.*, 1991). Although such procedures allow for the comparison of the phototoxicity of dyes after cell uptake, the procedure does not take into account the many important *in vivo* parameters which govern dye distribution and phototoxicity, including interactions with blood components (albumin or lipoproteins), capillary permeability in tumour, or distribution in interstitial tumour space (for a recent review see Paquette and van Lier, 1991*b*).

Using the *in vivo/in vitro* cell survival assay as described in this report, actual *in vivo* distribution and cell uptake of dye are respected, while an *in vitro* illumination allows accurate quantification of direct cell killing potential. EMT-6 cell survival curves (Figure 2) after *in vivo* administration of $10\,mg\,kg^{-1}$ P-II revealed that at the maximum fluence of 40 $J\,cm^{-2}$ only 20% of the EMT-6 cells were inactivated. However to elicit a complete *in vivo* tumour response with the same dye dose, a 10-fold higher fluence of 400 $J\,cm^{-2}$ was required, suggesting that at least at the top layer of the tumour, direct cell killing may contribute to tumour necrosis. Overall tumour cell survival after *in vivo* PDT with P-II has previously been shown to be high, this suggests a predominantly indirect action mechanism (Fingar & Henderson, 1987) which is in agreement with the high cell survival observed in our *in vivo/in vitro* test.

Unlike P-II, all three phthalocyanines tested in this study exhibited high potential for direct cell killing in PDT. *In vitro* photoinactivation of EMT-6 cells after *in vivo* administration of $10\,\mu mol\,kg^{-1}$ dye gave $LD_{90}$ varying from 15.7 $J\,cm^{-2}$ for $AlPcS_2$ through 5.2 and 6.4 $J\,cm^{-2}$ for $AlPcS_{2.4}P_{1.2}$ and $AlPcS_{3.2}P_{0.4}$ respectively (Figure 2). The phthalimidomethylated ALPcSP $LD_{90}$ values are approximatley half that of $AlPcS_2$ which parallels their relative photoactivities under *in vitro* conditions with Chinese hamster fibroblast V-79 cells (Paquette *et al.*, 1991*a*). In additon to passive diffusion, dye uptake by neoplastic cells *in vivo* can be mediated by endocytosis (Ben-Hur *et al.*, 1987; Roberts and Berns, 1989). These processes are modulated by dye interaction with constituents in interstitial liquid such as albumin, collagene or low density lipoproteins, which compete with dye uptake by neoplastic cells. On the other hand, binding of hydrophilic dye on low density lipoprotein is believed to favour specific endocytosis by neoplastic cells (Kessel *et al.*, 1987). Our data suggest that addition of phthalimidomethyl groups to AlPcS favours their localisation in neoplasmic cells, resulting in a two fold increase in direct cell killing and a 4-fold increase in the therapeutic window.

Comparison of the *in vivo* and *in vitro* phototoxicities (Paquette *et al.*, 1991*a*) of the AlPcS and AlPcSP suggest that the hydrophobic/amphiphilic properties of the dyes have less impact on the *in vivo* as compared to the *in vitro* activities. In the V-79 cell survival assay, the more hydrophobic and amphiphilic $AlPcS_{2.4}P_{1.4}$ fraction was over 3-fold more photoactive than the hydrophylic $AlPcS_{3.2}P_{0.4}$ fraction. In the *in vivo/in vitro* EMT-6 cell survival assay, both dye preparations exhibited similar direct cell killing potentials. This suggests the futility of further attempts to increase the hydrophobic/amphiphilic character of these photosensitisers in order to improve their direct cell killing potential during *in vivo* PDT.

The degree of sulfonation of AlPcS strongly effects the

**Table I** Photodynamic activity on EMT-6 tumour

| *Sensitiser* | *Minimum dose required for 100% tumour cure*[a] |
|---|---|
| $AlPcS_2$ | $0.5\,\mu mol\,kg^{-1}$ ($0.38\,mg\,kg^{-1}$) |
| $AlPcS_4$ | $5.0\,\mu mol\,kg^{-1}$ ($4.8\,mg\,kg^{-1}$) |
| $AlPcS_{3.2}P_{0.4}$ | $0.5\,\mu mol\,kg^{-1}$ ($0.47\,mg\,kg^{-1}$) |
| $AlPcS_{2.4}P_{1.2}$ | $0.5\,\mu mol\,kg^{-1}$ ($0.5\,mg\,kg^{-1}$) |
| Photofrin II[tm] | $10\,mg\,kg^{-1}$ |

[a] 24 h after injection of photosensitiser, tumour bearing BALB/c mice were illuminated at a fluence of 400 $J\,cm^{-2}$. An initial necrosis appeared within 48 h post-treatment and was followed to cure.

**Table II** Therapeutic window

| | *Dose*[b] *in* $\mu mol\,kg^{-1}$ | | | | |
|---|---|---|---|---|---|
| *Sensitiser*[a] | *0.5* | *1.0* | *2.5* | *5.0* | *10* |
| $AlPcS_2$ | Min. cure dose | Cure + slight oedema | Lethal dose | | |
| $AlPcS_{3.2}P_{0.4}$ | Min. cure dose | Cure + slight oedema | Cure + paralysis of leg | Lethal dose | |
| $AlPcS_{2.4}P_{1.2}$ | Min. cure dose | Cure | Cure | Cure + slight oedema | Lethal dose |

[a] Experiments with Photofrin II gave a lethal dose of $20\,mg\,kg^{-1}$. [b] 24 h after injection of photosensitiser, tumour bearing BALB/c mice were illuminated at a fluence of 400 $J\,cm^{-2}$. An initial necrosis appeared within 48 h post-treatment and was followed to cure.

minimal drug dose required for tumour response. Thus, $AlPcS_2$ is ten times more photoactive than the corresponding $AlPcS_4$ (0.5 μmol $kg^{-1}$ and 5 μmol $kg^{-1}$, respectively), confirming that the increase in hydrophobic/amphiphilic character of AlPcS improves the effective overall distribution of the dye in the tumour. This assumption is in agreement with recent observations by Peng *et al.* (1990*a*,*b*) showing that $AlPcS_2$ at 24 h post-injection, mainly localises in the neoplastic cells of LOX tumour bearing mice. In contrast, the $AlPcS_4$ remained mainly in the stroma, even at 48 h post-injection. It is likely that the highly hydrophilic character of $AlPcS_4$ contributes to strong binding to the protein component in the stroma, while the amphiphilic nature of $AlPcS_2$ promotes interaction with the plasma membrane of neoplastic cells. Adding lipophilic phthalimidomethyl substituents to AlPcS did not significantly improve the required dye dose for 100% tumour cure. Both $AlPcS_{2.4}P_{1.4}$ and $AlPcS_{3.2}P_{0.4}$ fractions required, in our EMT-6 model, the same minimal dye dose for tumour cure as $AlPcS_2$ (0.5 μmol $kg^{-1}$). It thus appears that the inclusion of hydrophobic phthalimidomethyl groups in AlPcS has a beneficial PDT effect in that it outweighs the expected decrease in activity resulting from higher sulphonation levels (Brasseur *et al.*, 1987*a*).

Extrapolation of the high direct cell killing potential of the AlPcSP to the mechanisms of the PDT response of the EMT-6 tumour, should be done with caution. Although the minimal injected dye dose for tumour response was 20-fold lower than the dye dose employed in the *in vivo*/*in vitro* cell survival assay (0.5 and 10 μmol $kg^{-1}$, respectively), the fluence in the *in vivo* PDT assay was at least 60-fold higher than that required for a 90% cell inactivation in the *in vivo*/*in vitro* assay. Furthermore, absorption of red light by the tumour tissue allows for only about 20% of incident light to reach the lower part of tumour (Henderson, 1989). These factors combined suggest that the deeper seated part of the EMT-6 tumour will receive somewhat lower combined light/dye doses than the $LD_{90}$ values obtained in the *in vivo*/*in vitro* cell killing assay. Thus it is likely that in the deeper seated parts of the tumour, and at the lower effective dye doses, tumour cure will involve indirect mechanism for cell killing.

Indirect tumour cell killing after PDT has been related to the release of vasoactive agents including histamine and prostaglandins, and this, in addition to local effects, could trigger also substantial systemic toxicity (Fingar *et al.*, 1991; Kerdel *et al.*, 1987; Lim *et al.*, 1986). Accordingly, we evaluated the therapeutic window in our tumour model, i.e. the range of the minimal dye doses required for 100% tumour cure and the dose which induced systemic toxicity (Table II). Under our experimental conditions both $AlPcS_2$ and $AlPcS_{3.2}P_{0.4}$ proved highly phototoxic at 5.0 μmol $kg^{-1}$ suggeting that, although these compounds have a good potential for direct cell killing, they also localise in sites responsible for the release of inflammatory agents. Among the three sensitisers which exhibit photodynamic action at 0.5 μml $kg^{-1}$, the $AlPcS_{2.4}P_{1.2}$ has the largest therapeutic window. This suggests that the addition of the phthalimidomethyl group on amphiphilic $AlPcS_2$ reduces unwanted dye interaction with cells capable of releasing vasoactive agents (e.g. mast cells).

In conclusion, these results imply a subtle balance between the hydrophobic and the amphiphilic characteristics of phthalocyanine sensitisers and their action mechanisms, with important implications in terms of both photodynamic potency and deleterious effects. The addition of the hydrophobic phthalimidomethyl groups on lower sulphonated AlPcS should improve the PDT outcome by increasing direct tumour cell killing while limiting deleterious effects on normal surrounding tissue. In the EMT-6 tumour model, the current class of phthalocyanines induces a higher direct cell killing and tumour response as compared to P-II. Further chemical modifications should be aimed at improving the therapeutic window, which would allow the administration of higher doses of dye and red light, resulting in augmented direct cell killing and cure of malignant tumours.

This work was supported by the Medical Research Council of Canada. The authors thank Huguette Savoie and Gloria Cheal for expert technical assistance.

### References

ALI, H., LANGLOIS, R., WAGNER, J.R., BRASSEUR, N., PAQUETTE, B. & VAN LIER, J.E. (1988). Biological activities of phthalocyanines X. Syntheses and analyses of sulfonated phthalocyanines. *Photochem. Photobiol.*, **44**, 713.

BARR, H., TRALAU, C.J., BOULOS, P.B. & 4 others (1990). Selective necrosis in dimethylhydrazine-induced rat colon tumours using phthalocyanine photodynamic therapy. *Gastroenterology*, **98**, 1532.

BEN-HUR, E., SIWECKI, J.A., NEWMAN, H.C., CRANE, S.W. & ROSENTHAL, I. (1987). Mechanism of uptake of sulfonated metallophthalocyanines by cultured mammalian cells. *Cancer Let.*, **38**, 215.

BRASSEUR, N., ALI, H., LANGLOIS, R. & VAN LIER, J.E. (1987*a*). Biological activities of phthalocyanines – VII. Photoinactivation of V-79 Chinese hamster cells by selectively sulfonated gallium phthalocyanines. *Photochem. Photobiol.*, **46**, 739.

BRASSEUR, N., ALI, H., LANGLOIS, R., WAGNER, J.R. & VAN LIER, J.E. (1987*b*). Biological activities of phthalocyanines – V. Photodynamic therapy of EMT-6 mammary tumors in mice with sulfonated phthalocyanines. *Photochem. Photobiol.*, **45**, 581.

CHAN, W.S., SVENSEN, R., PHILLIPS, D. & HART, I.R. (1986). Cell uptake, distribution, and response to aluminium chloro sulphonated phthalocyanine, a potential anti-tumour photosensitizer. *Br. J. Cancer*, **53**, 255.

CHATLANI, P.T., BEDWELL, J., MACROBERT, A.J. & 5 others (1991). Comparison of distribution and photodynamic effects of di- and tetra-sulphonated aluminium phthalocyanines in normal rat colon. *Photochem. Photobiol.*, **53**, 745.

DOUGHERTY, T.J. (1987). Photosensitizers: therapy and detection of malignant tumors. *Photochem. Photobiol.*, **45**, 897.

FERRARIO, A. & GOMER, C.J. (1990). Systemic toxicity in mice induced by localized porphyrin photodynamic therapy. *Cancer Res.*, **50**, 539.

FINGAR, V.H. & HENDERSON, B.W. (1987). Drug and light dose dependence of photodynamic therapy: a study of tumor and normal tissue response. *Photochem. Photobiol.*, **46**, 837.

FINGAR, V.H., WIEMAN, T.J. & WEBER DOAK, K. (1991). Changes in tumor interstitial pressure induced by photodynamic therapy. *Photochem. Photobiol.*, **53**, 763.

HENDERSON, B.W. & BELLNIER, D.A. (1989). Tissue localization of photosensitizers and the mechanism of photodynamic tissue destruction. In *Photosensitizing Compounds: Their Chemistry, Biology and Clinical Use* (Ciba Foundation Symposium; Vol. 146). John Wiley & Sons: New York.

HENDERSON, B.W. (1990). The significance of vascular photosensitization in photodynamic therapy. In *Future Directions and Applications in Photodynamic Therapy*, Gomer, C.J. (ed.), p. 153. SPIE Institutes for Advanced Optical Technologies. Vol. IS 6. Bellingham: Washington.

JOCHAM, D., BEER, M., BAUMGARTNER, R., STAEHLER, G. & UNSOLD, E. (1989). Long-term experience with integral photodynamic therapy of TIS bladder carcinoma. In *Photosensitizing Compounds: Their Chemistry, Biology and Clinical Use*. Bock, G. & Harnell (eds), P. 198, Ciba Foundation Symposium, 146, Wiley: Chichester.

KERDEL, F.A., SOTER, N.A. & LIM, H.W. (1987). *In vivo* mediator release and degranulation of mast cells in hematoporphyrin derivative-induced phototoxicity in mice. *J. Invest. Dermatol.*, **88**, 277.

KESSEL, D. (1984). Hematoporphyrin and HPD: photophysics, photochemistry and phototherapy. *Photochem. Photobiol.*, **39**, 851.

KESSEL, D., THOMPSON, P., SAATIO, K. & NANTWI, K.D. (1987). Tumor localization and photosensitization by sulfonated derivatives of tetraphenylporphine. *Photochem. Photobiol.*, **45**, 787.

LIM, H.W., PARKER, D. & MARCUS, A.J. (1986). Generation of eicosanoids from mast cells exposed to protoporphyrin and irradiation. *Clin. Res.*, **34**, 763R.

MOAN, J. (1986). Porphyrin photosensitization and phototherapy. *Photochem. Photobiol.*, **43**, 681.

PAQUETTE, B., ALI, H., LANGLOIS, R. & VAN LIER, J.E. (1988). Biological activities of phthalocyanines VIII. Cellular distribution in V-79 Chinese hamster cells and phototoxicity of selectively sulfonated aluminium phthalocyanines. *Photochem. Photobiol.*, **47**, 215.

PAQUETTE, B., BOYLE, R.W., ALI, H., MCLENAN, A.M., TRUSCOTT, T.G. & VAN LIER, J.E. (1991*a*). Sulfonated phthalimidomethyl aluminium phthalocyanine: The effect of hydrophobic substituents on the in vitro phototoxicity of phthalocyanines. *Photochem. Photobiol.*, **53**, 323.

PAQUETTE, B. & VAN LIER, J.E. (1991*b*). Phthalocyanines and related compounds: structure-activity relationships. In *Photodynamic Therapy: Basic Principles and Clinical Aspects*, Dougherty, T.J. & Henderson, B.W. (eds). Marcel Dekker (in press).

PENG, Q., MOAN, J., FARRANTS, G., DANIELSEN, H.E. & RIMINGTON, C. (1991). Localization of potent photosensitizers in human tumor LOX by means of laser scanning microscopy. *Cancer Lett.*, **58**, 17.

PENG, Q., MOAN, J., NESLAND, J.M. & RIMINGTON, C. (1990*a*). Aluminium phthalocyanines with asymmetrical lower sulfonation and with symmetrical higher sulfonation: a comparison of localizing and photosensitizing mechanism in human tumor LOX xenografts. *Int J. Cancer*, **46**, 719.

PENG, Q., NESLAND, J.M., MOAN, J., EVENSEN, J.F., KONGSHANG, M. & RIMINGTON,C. (1990*b*). Localization of fluorescent Photofrin II and aluminium phthalocyanine tetrasulfonate in transplanted human malignant tumor LOX and normal tissues of nude mice using highly light-sensitive video intensification microscopy. *Int. J. Cancer*, **45**, 972.

REED, M.W.R., WIEMAN, T.J., SCHUSCHKE, D.A., TSENG, M.T. & MILLER, F.N. (1989). A comparison of the effects of photodynamic therapy on normal and tumor blood vessels in the rat microcirculation. *Radiation Res.*, **119**, 542.

ROBERTS, W.G. & BERNS, M.W. (1989). *In vitro* photosensitization 1: Cellular uptake and subcellular localization of mono-L-aspartyl chlorin $e_6$, chloro-aluminium sulfonated phthalocyanine, and Photofrin II. *Lasers in Surg. & Med.*, **9**, 90.

ROSENTHAL, I. (1991). Phthalocyanines as photodynamic sensitizers. *Photochem. Photobiol.*, **53**, 859.

SELMAN, S.H., KREIMER-BIRNBAUM, M., KLAUNIG, J.E., GOLDBLATT, P.J., KECK, R.W. & BRITTON, S.L. (1985). Blood flow in transplantable tumors treated with hematoporphin derivative and light. *Cancer Res.*, **45**, 1924.

STAR, W.M., MARUNISSEN, H.P.A., VAN DEN BERG BLOK, A.E., VERSTEEG, J.A.C., FRANKEN, K.A.P. & REINHOLD, H.S. (1986). Destruction of rat mammary tumor and normal tissue microcirculation by hematorphin derivative photoradiation observed *in vivo* in sandwich observation chambers. *Cancer Res.*, **46**, 2532.

TRALAU, C.J., BARR, H., SANDERMAN, D.R., BARTON, T., LEWIN, M.R. & BOWN, S.G. (1987). Aluminium sulfonated phthalocyanine distribution in rodent tumors of the colon,brain and pancreas. *Photochem. Photobiol.*, **46**, 777.

VAN LIER, J.E. & SPIKES, J.D. (1989). The chemistry, photophysics and photosensitizing properties of phthalocyanines. In *Photosensitizing Compounds: Their Chemistry, Biology and Clinical Use*. Bock, G. & Harnell (eds), P. 17, Ciba Foundation Symposium, 146, Wiley: Chichester.

VAN LIER, J.E. (1990). Phthalocyanines as sensitizers for PDT of cancer. In *Photodynamic Therapy of Neoplastic Disease*. Vol. 1, Kessel, D. (ed.), p. 279, CRC Press: Boca Raton, FL.